# Clinical impact of idiopathic pulmonary fibrosis on SARS-CoV-2 patient outcomes: a comprehensive analysis in the pre-vaccination era

**DOI:** 10.3389/fmed.2025.1567232

**Published:** 2025-05-21

**Authors:** Omar Tamimi, Zeenat Safdar, Nadia Siddiqui, Tariq Nisar, Deepa Gotur

**Affiliations:** ^1^Department of Internal Medicine, Houston Methodist Hospital, Houston, TX, United States; ^2^Houston Methodist Lung Center, Houston, TX, United States; ^3^Division of Pulmonary Critical Care, Houston, TX, United States; ^4^Jinnah Medical and Dental College, Karachi, Pakistan; ^5^Center for Health Data Science and Analytics, Houston Methodist, Houston, TX, United States

**Keywords:** COVID-19, idiopathic pulmonary fibrosis, outcomes, viral infections, hospitalization, length of stay, hospitalization cost

## Abstract

**Introduction:**

Our retrospective study aimed to evaluate the impact of idiopathic pulmonary fibrosis (IPF) on the clinical outcomes of COVID-19 admissions using data from the 2020 nationwide inpatient sample (NIS).

**Methods:**

We performed multivariate adjustment for baseline comorbidities and demographics after univariate screening.

**Results:**

Among the 1,018,915 adults hospitalized with COVID-19 in 2020, 910 were also diagnosed with IPF. Patients admitted with both COVID-19 and IPF had a higher risk of mortality compared to those without IPF [adjusted OR 1.87 (95% CI 1.13-2.70), *p* < 0.01]. Additionally, patients with both conditions had higher odds of requiring mechanical ventilation [adjusted OR 1.66 (95 % CI 1.13–2.42) *p* = 0.01] and needing mechanical ventilation within the first 24 h of admission [adjusted OR 1.87 (95% CI 1.013–3.39) *p* = 0.04]. IPF patients incurred higher mean total hospitalization charges [$140,790 vs. $79,045, adjusted difference + $60,577 (SD ± 52,460)] and had a longer mean length of stay [11.2 vs. 7.5 days, adjusted difference 3.3 days longer (SD ± 2.0)] compared to the non-IPF cohort (*p* = 0.02).

**Discussion:**

Our findings suggest that IPF significantly worsens the clinical outcomes of COVID-19 hospitalizations, leading to increased healthcare utilization and costs. Further studies are needed to study this subpopulation during the postvaccination era to understand the effects on patient outcomes and to explore potential targeted interventions for improving prognosis in patients with both COVID-19 and IPF.

## Introduction

Idiopathic Pulmonary Fibrosis (IPF) is a rare, chronic interstitial lung disease (ILD) of unknown origin with a progressive phenotype. Prevailing theories suggest that repeated subtle injuries to genetically predisposed alveoli lead to failure of alveolar re-epithelization, leading to a cascade of inflammation, cytokine release, and collagen deposition, ultimately ending in irreversible injury and scarring of lung tissue (1).

The estimated incidence rates of IPF per 10,000 individuals ranged from 0.35 to 1.30 in Asia-Pacific nations, 0.09–0.49 in Europe, and 0.75–0.93 in North America (2). IPF has a poor prognosis, with currently approved therapies slowing disease progression but failing to reverse it, resulting in a median survival rate of about 3 years (3). Previous studies observed similarities in gene expression patterns, prognostic markers, and dysfunctional alveolar type II (AT2) cellular processes leading to fibrosis in severe COVID-19 and IPF (1). Both diseases share fundamental immune and alveolar responses, corresponding to epidemiological similarities, such as affecting older adults, having a higher incidence in males, and poor outcomes. The severity and mortality rate of ILD can be exacerbated by viral infections like COVID-19, particularly in patients with poor lung function (4, 5). Our objective was to evaluate the influence of an IPF diagnosis on COVID-19 admissions during the pre-vaccination era, utilizing data from the National Inpatient Sample database.

## Materials and methods

This retrospective study utilized the 2020 National Inpatient Sample (NIS) dataset from the Agency for Healthcare Research and Quality (AHRQ), on hospitalizations from January 1, 2020, to December 31, 2020 (6). All patients aged 18 years and older who were non-electively admitted to the hospital with a principal diagnosis of COVID-19 were included. The International Classification of Diseases, 10th clinical modification (ICD-10-CM) codes were used to identify patients’ co-morbid conditions, and ICD-10 procedure codes were used to identify procedures. We followed all AHRQ guidelines^1^ for reporting and were exempt from IRB review as the National inpatient sample is a publicly available deidentified dataset.

### Covariates

The NIS database contains detailed information regarding admissions and provides data for in-hospital diagnoses, procedures, and outcomes. We divided COVID-19 principal admissions into those patients who also had idiopathic pulmonary fibrosis (IPF) (ICD-10-CM code J84.112). Patient demographics were considered, including age, race, sex, Charlson co-morbidity index, insurance status, median household income, and hospital region/size/teaching status. We also accounted for co-morbidities such as hypertension, diabetes mellitus (type 1, 2 or other), coronary artery disease (CAD), chronic obstructive pulmonary disease (COPD), morbid obesity, chronic kidney disease (CKD, stage 1–5), and end-stage renal disease (ESRD). Procedures identified during the index admission included mechanical ventilation (both non-invasive positive pressure ventilation (NIPPV) and invasive positive-pressure ventilation (IPPV) and vasopressors. A complete list of ICD-10 codes used can be found in Supplementary Appendix.

### Study outcomes

The primary outcome was in-hospital mortality. Secondary outcomes included mechanical ventilation, vasopressor use, mean length of stay, and mean total hospitalization charges.

### Statistical methods

Statacorp LLC, College Station, TX, known as STATA^®^ version 17 was used for statistical analysis. The unweighted sample comprised 6.5 million observations, while the weighted sample was approximately 32.4 million discharges for the year 2020. Patient data with a principal diagnosis of COVID-19 were retrieved using ICD-10-CM codes, and this group was further subdivided based on the presence or absence of comorbid IPF. Missing data in continuous variables were replaced using mean value imputation. Following imputation, propensity score matching using K-Nearest Neighbor (KNN) with a 1:4 ratio was applied to compare COVID-19 patients with and without IPF. The matching process achieved good covariate balance, indicated by an average standardized mean difference (SMD) of less than 0.10 and a maximum SMD of 0.08, both within acceptable balance thresholds. A survey design was implemented using the NIS stratum, hospital IDs, and discharge weights (DISCWT). Weighted *t*-test were used to identify differences between continuous variables and weighted chi-squared χ^2^ tests were used for categorical variables. Weighted logistic regression models were constructed for binary outcomes and weighted generalized linear models were used for continuous outcomes. A univariate screen was performed for each variable, and variables that met the threshold of significance were then entered into the multivariate regression model. A multivariate logistic regression analysis was used to estimate the probability of primary and secondary outcomes while controlling for the other independent variables. Statistical significance was established at a two-tailed *p*-value of less than 0. 05. We reported odds ratios (OR) and 95% Confidence intervals (CI). All analyses were performed using R version 4.1.3 and STATA Version 17.

## Results

### Demographics and baseline comorbidities

From January 1 to December 31, 2020, there were 32,355,827 discharges. Of these, 1,018,915 patients with a principal diagnosis of COVID-19 met the inclusion criteria (18 years or older, non-elective admission) and were hospitalized during this study period. Among these COVID admissions, 910 had a co-diagnosis of IPF (<1%).

Compared to COVID-19 patients without IPF, those in the IPF cohort were less likely to be female (36.8% vs. 47.2%, *p* < 0.01), were older (72 years vs. 65 years, *p* < 0.01), had a higher proportion of Caucasians (69.3% vs. 52.4, *p* < 0.01), and a higher proportion of Medicare beneficiaries (71.7% vs. 54.9%, *p* < 0.01). There was no statistically significant difference between the two groups in the distribution of hospital region (*p* = 0.09), hospital bed size (*p* = 0.06), or hospital teaching status (*p* = 0.15) (Table 1).

**TABLE 1:** COVID-19 and idiopathic pulmonary fibrosis (IPF) patient-level characteristics.

Characteristics	COVID-19 without IPF (%)	COVID-19 with IPF (%)	*p*-value
***N* = 1,018,915**	***N* = 1,018,005 (>99%)**	***N* = 910 (<1%)**	
Sex (female)	47.2	36.8	0.01
Mean age years (SD)	65	72	<0.01
**Race**			<0.01
Caucasians	52.4	69.3	
African American	18.6	6.3	
Hispanics	20.6	16.5	
Asian or Pacific Islander	3.3	3.4	
Native American	1.0	4.0	
Others	4.1	0.6	
**Median household income**			0.02
<$49,999	33.9	27.9	
$50,000–$64,999	27.6	36.9	
$65,000–$85,999	22.2	22.9	
>$86,000	16.4	12.3	
**Insurance status**			<0.01
Medicare	54.9	71.7	
Medicaid	12.3	6.1	
Private	29.1	20.0	
Self-pay	3.6	2.2	
**Hospital region**			0.09
Northeast	18.0	18.7	
Midwest	23.2	30.8	
South	41.5	36.3	
West	17.4	14.3	
**Hospital bed size**			0.06
Small	25.2	24.2	
Medium	29.0	22.0	
Large	45.8	53.9	
**Hospital teaching status**			0.15
Rural	10.8	12.6	
Urban non-teaching	19.3	13.7	
Urban teaching	69.8	73.6	
**Comorbidities**			
Hypertension	67.9	71.4	0.32
Coronary artery disease	18.3	31.3	<0.01
Chronic obstructive pulmonary disease	12.9	23.6	<0.01
Diabetes mellitus	40.4	41.8	0.71
Morbid obesity	19.0	8.2	<0.01
Chronic kidney disease (stage 1–5)	11.0	6.0	0.03
End-stage renal disease	3.7	1.1	0.06
**Charlson co-morbidity index**			<0.01
0	27.7	16.5	
1	27.9	25.3	
2	16.3	22.5	
≥3	28.1	35.7	

Regarding baseline co-morbidities, patients with COVID-19 and IPF had a higher proportion of co-morbid COPD (23.6% vs. 12.9%, *p* < 0.01), CAD (31.3% vs. 18.3%, *p* < 0.01) and a Charlson co-morbidity index of at least 3 (35.7% vs. 28.1%, *p* < 0.01) indicating higher patient complexity and increased number of co-morbidities in this cohort compared to those with COVID-19 without IPF (Table 1). However, the IPF cohort had lower proportions of co-morbid CKD (6.0% vs. 11.0%, *p* = 0.03) and morbid obesity (8.2% vs. 19.0%, *p* < 0.01) compared to the non-IPF cohort. There was no statistically significant difference between the two groups in the prevalence of hypertension (67.9% vs. 71.4%, *p* = 0.32), diabetes mellitus (40.4% vs. 41.8%, *p* = 0.71), or ESRD (3.7% vs. 1.1%, *p* = 0.06). Table 1 highlights all baseline demographics and co-morbidities between both cohorts.

### In-hospital mortality

**FIGURE 1:**
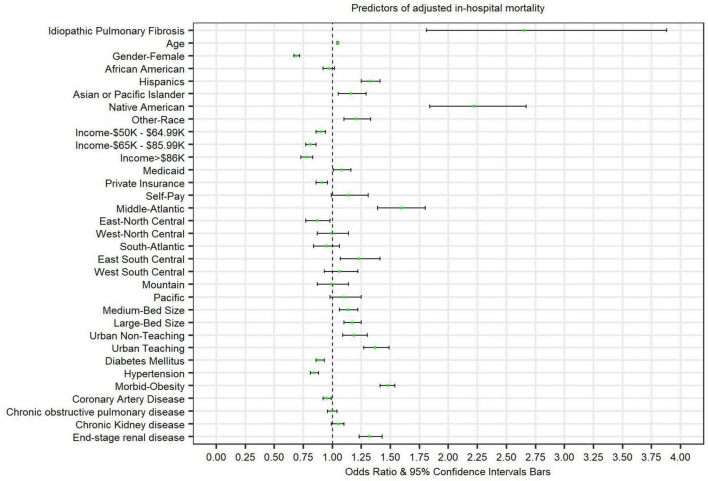
Forest plot presenting predictors of adjusted in-hospital mortality in patients admitted with COVID-19.

There were 113,180 (11.1%) total in-hospital COVID-19 deaths. Among patients with both COVID-19 and IPF, 270 (29.7%) died during the study period. After multivariate adjustment, patients admitted with COVID-19 and IPF were at higher odds for in-hospital mortality [adjusted OR: 1.87 (95% CI 1.13–2.70) *p* = < 0.01] compared to patients without IPF. Table 2 and Figure 1 highlight independent predictors of adjusted in-hospital mortality for COVID-19 admissions including age, male gender, Hispanic, Asian/Pacific Islander, Native American, or other races (compared to Caucasians), lower median household incomes (below $49,000), insurance status, hospital location, morbid obesity, or ESRD.

**TABLE 2:** Predictors of adjusted in-hospital mortality in patients admitted with COVID-19.

Characteristics	Mortality (OR) [95% CI]	*p*-value
Idiopathic pulmonary fibrosis	1.87 [1.13–2.70]	<0.01
Age	1.05 [1.04–1.05]	<0.01
Sex (female)	0.69 [0.67–0.72]	<0.01
**Race**		
Caucasian	Ref	
African American	0.97 [0.92–1.02]	0.21
Hispanics	1.33 [1.25–1.41]	<0.01
Asian or Pacific Islander	1.16 [1.05–1.29]	<0.01
Native American	2.22 [1.84–2.67]	<0.01
Others	1.21 [1.10–1.33]	<0.01
**Median household income**		
<$49,000	Ref	
$50,000–$64,999	0.90 [0.86–0.94]	<0.01
$65,000–$85,999	0.81 [0.77–0.86]	<0.01
>$86,000	0.78 [0.73–0.83]	<0.01
**Insurance status**		
Medicare	Ref	
Medicaid	1.08 [1.01–1.16]	<0.03
Private	0.91 [0.86–0.96]	<0.01
Self-pay	1.14 [0.99–1.31]	0.06
**Hospital division**		
New England	Ref	
Middle Atlantic	1.59 [1.39–1.80]	<0.01
East North Central	0.87 [0.77–0.98]	<0.03
West North Central	0.99 [0.87–1.14]	0.99
South Atlantic	0.95 [0.84–1.06]	0.35
East South Central	1.23 [1.07–1.41]	<0.01
West South Central	1.06 [0.93–1.22]	0.37
Mountain	0.99 [0.87–1.14]	0.91
Pacific	1.10 [0.98–1.25]	0.11
**Hospital bedside**		
Small	Ref	
Medium	1.14 [1.06–1.22]	<0.01
Large	1.17 [1.10–1.25]	<0.01
**Hospital teaching status**		
Rural	Ref	
Urban non-teaching	1.19 [1.09–1.30]	<0.01
Urban teaching	1.37 [1.27–1.49]	<0.01
**Comorbidities**		
Diabetes mellitus	0.89 [0.86–0.93]	<0.01
Hypertension	0.84 [0.81–0.88]	<0.01
Morbid obesity (BMI > 35)	1.48 [1.41–1.54]	<0.01
Coronary artery disease	0.95 [0.92–0.99]	<0.01
Chronic obstructive pulmonary disease	0.99 [0.96–1.04]	0.98
Chronic kidney disease (stage 1–5)	1.05 [0.99–1.10]	0.06
End-stage renal disease	1.32 [1.23–1.43]	<0.01

### In-hospital complications

#### Mechanical ventilation

**FIGURE 2:**
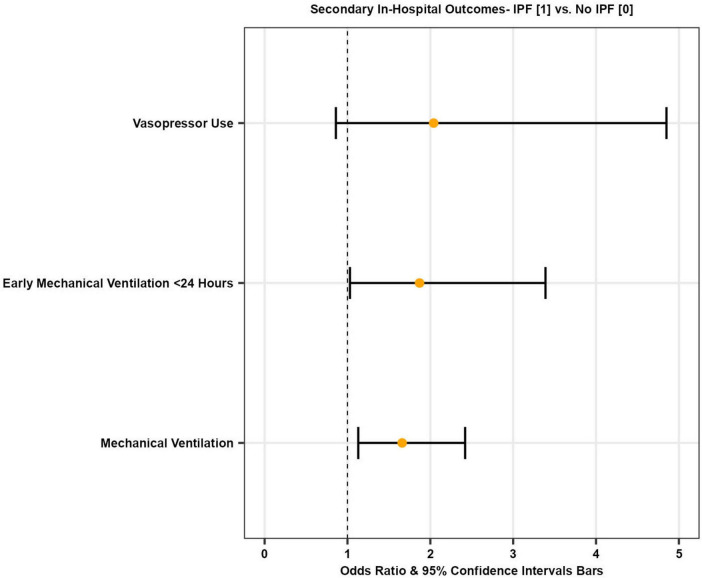
Forest plot illustrating odds of secondary in-hospital outcomes in patients with COVID-19 with and without IPF.

Overall, 13.7% of patients admitted with COVID-19 required mechanical ventilation (both invasive (IPPV) and non-invasive (NIPPV) positive pressure ventilation). Among COVID-19 admissions with co-morbid IPF, 25.8% of patients required mechanical ventilation. After multivariate adjustment, IPF patients admitted with COVID-19 had higher odds of requiring mechanical ventilation compared to those without IPF [adjusted OR 1.66 (95% CI 1.13–2.42), *p* = 0.01] (Figure 2 and Table 3).

**TABLE 3:** Odds of secondary in-hospital outcomes in patients with COVID-19 adjusted for age, gender, race, median income, Charlson index, hospital region, teaching status, hospital size, insurance status, and baseline co-morbidities.

Outcome	IPF (OR)	95% CI	*p*-value
Mechanical ventilation	1.66	[1.13–2.42]	<0.01
Early Mechanical Ventilation (< 24 h of admission)	1.87	[1.03–3.39]	0.04
Vasopressor use	2.04	[0.86–4.85]	0.10

[] refers to a 95% confidence interval.

#### Early mechanical ventilation

The mean time from admission to mechanical ventilation for COVID-19 patients was 4.1 days, while it was 5.2 days for IPF patients with COVID-19. Early mechanical ventilation is defined as requiring mechanical ventilation within 24 h of the index admission. After multivariate adjustment, there was a statistically significant increase in the odds of early mechanical ventilation in IPF patients with COVID-19 compared to COVID-19 patients without IPF [adjusted OR 1.87 (95% CI 1.03–3.39), *p* = 0.04] (Figure 2 and Table 3).

#### Vasopressor use

A total of 18,475 (1.8%) COVID-19 patients required vasopressors during hospitalization. Among those with both COVID-19 and IPF, 4.4% required vasopressors. However, after multivariate adjustment, there was no statistically significant difference in the odds of requiring vasopressors during hospitalization between the two cohorts [adjusted OR 2.04 (95% CI 0.86–4.85), *p* = 0.10] (Figure 2 and Table 3).

#### In-hospital quality measures

##### Length of stay

The mean length of stay (LOS) for patients admitted with COVID-19 in 2020 was 7.5 days. For patients with IPF, the mean LOS was 11.2 days. After multivariate adjustment, the IPF cohort had a statistically significant longer mean LOS [adjusted difference 3.3 days longer, (SD ± 2.0), *p* = < 0.01] (Table 4).

**TABLE 4:** Other secondary in-hospital outcomes.

Outcome	COVID without IPF	COVID with IPF	*p*-value
Mean total hospitalization charges ($)	79,045 [SD ± 2,240]	140,790 [SD ± 50,567]	
Adjusted difference in total charges ($)	Ref	+ 60,577 [SD ± 52,460]	0.02
Mean length of stay (days)	7.48 [SD ± 0.07]	11.23 [SD ± 1.9]	
Adjusted difference in length of stay (days)	Ref	3.29 days more [SD ± 2.04]	<0.01

##### Total hospitalization charges

Patients admitted with COVID-19 in 2020 had a mean total hospitalization charge of $79,045. Those with both COVID-19 and IPF had a mean total hospitalization charge of $140,790. Compared to COVID-19 admissions without IPF, those with IPF had significantly higher adjusted total hospitalization charges [adjusted difference + $60,577, (SD ± 52,460), *p* = 0.02] (Table 4).

## Discussion

Our study revealed mostly similar baseline demographics between the two patient cohorts. However, patients in the COVID-IPF cohort were older and had a higher proportion of males and Caucasians. They also had a higher proportion of co-morbid COPD and CAD and a higher Charlson co-morbidity index but showed no differences in other baseline co-morbidities. After multivariate adjustment for all baseline demographics and co-morbidities, patients admitted to the hospital with a principal diagnosis of IPF and COVID-19 had higher odds of in-hospital mortality, mechanical ventilation, early mechanical ventilation, mean length of stay, and total hospitalization charges compared to those without IPF.

It is interesting to note that in our analysis, medium and large-sized hospitals as well as urban hospitals and teaching hospitals had higher odds of mortality compared to small hospitals and rural hospitals, respectively. This observation is multifactorial and may reflect differences in patient populations, hospital referral patterns, and illness severity that we hypothesize lead to higher case-mix index. The observed protective association of hypertension, CAD, and diabetes with mortality is noteworthy and likely reflects several limitations inherent to claims-based datasets. First, comorbidities captured in administrative data are dependent on accurate and complete coding. In critically ill patients, more severe and reimbursable diagnoses—such as disseminated intravascular coagulation (DIC), septic shock, or multiorgan failure—may be prioritized in coding, while chronic conditions like hypertension or diabetes may be underreported. As a result, patients lacking these comorbidities in the data may not necessarily be healthier but rather may reflect a sicker population whose chronic conditions were not coded, creating the appearance of a protective effect. Additionally, the presence of hypertension in claims data could, in some cases, reflect episodes of in-hospital hypertension. In this context, elevated blood pressure may serve as a surrogate for preserved hemodynamic status; for example, a patient with hypertension is less likely to be in shock, which could explain the inverse association with mortality. We acknowledge that a key limitation of this study is the reliance on administrative claims data, which may not always align with clinical diagnoses determined through chart review or direct assessment. Nevertheless, we acknowledge that these specific findings contradict many clinical observations and should be interpreted with extreme caution due to data limitations.

It is worth noting that cancer was not included as an individual covariate due to its relatively low prevalence among patients with IPF and its limited direct association with IPF-related outcomes. Additionally, the Charlson Comorbidity Index—used in our model—accounts for cancer and other major comorbidities, reducing the risk of overfitting while maintaining appropriate confounder control.

Patients with ILD, including IPF, may have greater odds of fatality when infected with COVID-19, even when accounting for age, gender, and other health conditions (7). COVID-19, caused by coronavirus, often presents with flu-like symptoms but can lead to severe complications such as pneumonia and respiratory failure, sometimes resulting in death. A major challenge during the pandemic was the sudden surge in patients requiring hospitalization, mechanical ventilation, and intensive care (8).

The SARS-CoV-2 virus can initially cause acute lung injury (ALI), which may progress to acute respiratory distress syndrome (ARDS) in severe cases. In some patients, these conditions resolve as lung function returns to normal. However, certain instances of ALI and ARDS can evolve into a more severe condition known as post-COVID-19 pulmonary fibrosis, necessitating timely intervention and proper management. The interplay between viral load and the immune response is crucial for eliminating the virus. Subsequently, a pronounced immune response in the second phase can lead to an immune overreaction, precipitating ALI and ARDS (9, 10). Endothelial cells have been suggested to play a crucial role in initiating the cascade of inflammation in blood vessels by attracting immune cells, causing leaky vessels, increasing the likelihood of blood clots, and eventually resulting in decreased oxygen levels in the lungs of severe COVID-19 cases (11).

Our study highlights the potential impact of IPF in patients who have contracted COVID-19 during the pre-pandemic era, consistent with findings by Alrajhi (12). SARS-CoV-2 induces fibrosis both directly and indirectly by entering cells through ACE2 receptors and integrins (αvβ3, αvβ6), triggering a profibrotic cascade involving TGF-β, which promotes the formation of myofibroblasts and collagen resulting in scarring. Indirectly, SARs-CoV-2 damages alveolar epithelial cells by recruiting macrophages and generating inflammatory mediators like IL-6 and TNF-α (12).

These pathogenic processes contribute to adverse outcomes, and the presence of multiple comorbidities exacerbates the situation, leading to higher mortality rates. For patients with pre-existing IPF, these mechanisms significantly worsen their condition since their lungs are already prone to fibrosis, necessitating targeted therapeutic strategies.

The analysis supports the epidemiological pattern observed in patients with coexisting IPF and COVID-19, predominantly affecting older males. This data is substantiated by two articles indicating a mean age of 68.30 ± 12.0 and 65 ± 10, respectively, with male predominance of 56.97% and 71.7% (5, 13, 14). These studies reinforce the likelihood that older males are at a higher risk of being affected by this co-existing condition. It is important to note that our study was conducted from data in the pre-pandemic era and due to the emergence of new variants as well as vaccination this data cannot be entirely extrapolated.

Mechanical ventilation includes both invasive (IPPV) as well as non-invasive positive pressure ventilation (NIPPV). Our results indicate that patients with IPF and COVID-19 may experience more rapid respiratory deterioration, requiring earlier intervention with mechanical ventilation. They are nearly twice as likely to require early mechanical ventilation compared to those with COVID-19 alone.

A study by Herold et al. found that 13 out of the 40 recruited patients with COVID-19 experienced deteriorating health and required mechanical ventilation with a median duration from hospital admission to intubation of 2 days (15). In contrast, our study, with a larger sample size, found a median duration of 4.1 days from hospital admission to intubation. Another study by Kooistra et al. indicated that COVID-19 patients with pulmonary fibrosis (PF) had longer durations on ventilators, ICU stays, and higher mortality rates compared to those without PF. Survivors with PF also experienced prolonged mechanical ventilation and ICU stays compared to non-PF survivors (16). Cabrera-Benitez et al.’s study suggests that mechanical ventilation, especially when the lungs are excessively stretched, may contribute to fibrosis development in ARDS patients. Animal model studies indicate that mechanical ventilation can induce lung fibrosis by stimulating fibroproliferation (17).

Despite the challenges associated with prolonged mechanical ventilation in IPF patients, advancements in pharmacological interventions targeting the underlying mechanisms of fibrosis hold promise for preventing lung fibrosis in critically ill patients. Novel therapies like pirfenidone and nintedanib, used for pulmonary fibrosis, show potential for patients with COVID-19. Nintedanib inhibits intracellular tyrosine kinases and acts on multiple pro-angiogenic receptors, including vascular endothelial growth factor receptors (VEGFR), fibroblast growth factor receptors (FGFR), and platelet-derived growth factor receptors (PDGFR) (18). In contrast, pirfenidone inhibits cell death, reduces ACE receptor expression, decreases inflammation by inhibiting TGF-β1, and scavenges reactive oxidative species (19). These actions could protect lung cells, and additional research is needed to evaluate the efficacy of these medications for IPF patients with COVID-19.

The mean length of stay (LOS) for patients admitted with COVID-19 in 2020 was 7.5 days. However, our study found that patients with IPF had a longer average LOS of 11.2 days, demonstrating a significant statistical difference even after adjusting for other influential factors. Another study reported that 52% of patients with COVID-19 and IPF had a mean hospital LOS of 10 days and a median ICU length of stay of 8 days, further highlighting the association between LOS and increased mortality risk which was consistent with our findings (14). The extended LOS may be attributed to factors such as the duration of medication courses, ongoing testing and monitoring to assess disease progression, and the overall stability of the patient’s health.

Our analysis showed a higher in-hospital mortality rate of 29.7% among patients with both IPF and COVID-19 compared to those with COVID-19 alone. A similar study by Esposito et al. revealed that ILD patients who contracted COVID-19 had more than four times the fatality rate (33%) compared to patients without ILD, and were more likely to require hospitalization and intensive care unit (ICU) admission (4). Another study by Cilli et al. found that 13 out of 46 patients (28.2%) succumbed to COVID-19 complications (14). Both studies support the hypothesis that patients with IPF, whose compromised lung function results from excessive collagen accumulation and alteration in pulmonary interstitium due to various matrix proteins, are particularly vulnerable to COVID-19 (20). The virus exacerbates existing damage, leading to deterioration in outcomes like respiratory failure.

The Charlson Comorbidity Index (CCI) is a tool utilized to quantify an individual’s comorbidities and evaluate their prognosis. A higher CCI score represents a high risk of mortality, reflecting the clinical complexity of patients who are elderly and have multiple comorbidities (21). Patients with higher CCI scores are at an increased risk of developing severe COVID-19. Our study data indicates that COVID-19 patients, both with and without IPF, tend to have higher CCI scores (at least 3), but this risk is particularly pronounced in patients with IPF who also have COVID-19. Higher-risk patients spent a longer duration of mechanical ventilation and had extended hospital stays compared to low-risk patients, with all deceased individuals having ARDS and being on mechanical ventilation, as supported by Juan Guardela et al. (22).

Our study examined patients hospitalized with both COVID-19 and IPF, revealing a higher prevalence of comorbidities such as COPD (23.6% vs. 12.9%, *p* < 0.01), CAD (31.3% vs. 18.3%, *p* < 0.01), and a Charlson comorbidity index of at least 3 (35.7% vs. 28.1%, *p* < 0.01) compared to those with COVID-19 alone. However, a lower proportion of patients in the IPF cohort had co-morbid CKD (6.0% vs. 11.0%, *p* = 0.03) and morbid obesity (8.2% vs. 19.0%, *p* < 0.01) compared with COVID-19 without IPF. Cilli et al., in their relatively small but similar study, clarified that out of 46 patients, 42 individuals (91.3%), had at least one comorbidity (14). This finding is supported by Docherty et al., who found that chronic pulmonary conditions like COPD (18%), cardiac diseases like CAD (31%), and Chronic Kidney Disease (16%) were associated with higher mortality rates in hospital settings (23). Despite expectations, comorbidities such as hypertension, diabetes mellitus, or end-stage renal disease were not notably prevalent among individuals with both IPF and COVID-19.

While most studies corroborate the higher prevalence of these three comorbidities (24–26) an older study by Miyake et al. presents contradictory findings regarding the association with diabetes mellitus (27). A large population-based study observed that diabetic individuals had a lower prevalence of IPF compared to non-diabetic individuals who had passed away (28). Baig et al. found that diabetic IPF patients exhibited an unexpected protective effect on hospital mortality, though the precise role of diabetes in the prognosis of acute exacerbation of IPF remains unclear. Contrary to expectations, having diabetes appears to be associated with a reduced risk of death (29). Our study, with the largest sample size compared to these previous studies, offers valuable insights into which comorbidities individuals warrant extra caution in individuals contracting infectious diseases.

Previous research has shown that poor lung function and obesity increase the risk of death from COVID-19. The study by Drake et al. found that among 161 ILD patients, 129 were obese and obesity in these patients was significantly associated with a higher risk of death from COVID-19 (*p* = 0.001), surpassing the risk associated with obesity alone in patients without ILD (5). In obese individuals, fat accumulates in the lungs and airways, leading to numerous lipid droplets in the interstitium and macrophages. IPF, a restrictive lung disease, is further worsened by sarcopenic obesity. It damages alveolar epithelial type II cells (AT2), reducing cellular synthesis and surfactant production, and increasing interstitial collagen, which contributes to scarring. Additionally, fat buildup exacerbates the harmful effects of environmental factors such as smoking (30).

Given these findings, several considerations are crucial in managing patients with IPF. Firstly, minimizing non-essential healthcare-related contact is imperative to reduce the risk of exposure to infectious agents. This may involve optimizing the use of telemedicine or online appointments to limit in-person interactions in settings like laboratories and waiting rooms, where the transmission of contagious diseases like COVID-19 is anticipated. By implementing these precautionary measures, healthcare providers can prioritize the safety and well-being of IPF patients while ensuring continuity of care (31). Patients must also be guided to stay up to date with both COVID-19 vaccines and subsequent booster shots. A study conducted by Duong-Quy et al., recommended antifibrotic therapy for patients with chronic COVID-19 experiencing dyspnea. It is crucial to expedite diagnosis and treatment to lessen the severity of adverse or fatal outcomes, administering either Nintedanib or Pirfenidone based on individual susceptibility (32). Further prospective studies assessing COVID-19’s impact on the outcomes of IPF patients in the hospital setting are warranted, particularly in the post-vaccination era to evaluate its impact and guide further management recommendations.

## Limitations

Data for this study was gathered from the NIS, where diagnoses and outcomes rely on ICD-10 discharge codes rather than laboratory and imaging findings. This reliance may lead to some undetected cases in our study. Nevertheless, the extensive sample size may mitigate the impact of potential errors or missed codes. Additionally, the NIS lacks the capability to assess the severity of IPF or specify the medications used for management. Consequently, we are unable to subgroup patients based on objective severity indicators or treatment approaches, which could influence our findings. Moreover, since the NIS database uses hospital ICD-10-CM codes, it lacks data on the types of medications administered. Our study period was limited to 2020, and subsequent variants of the virus may exhibit different virulence compared to strains prevalent during that year. Lastly, as vaccination began after our study period ended, its impact on outcomes was not assessed, warranting further investigation.

Despite these limitations, our study has notable strengths. It features a robust sample size and, to our knowledge, represents the largest examination of hospitalized IPF patients with COVID-19 in the USA. The HCUP-NIS employs internal and external quality control measures to address potential errors and is a well-validated representation with strong generalizability to the US population.

## Conclusion

Our study underscores the importance of prioritizing IPF as a critical determinant of COVID-19 outcomes before widespread vaccination efforts. Particularly in individuals with elevated risks of mortality- such as those with advancing age, male gender, and comorbidities like COPD, CAD, CKD, and obesity- there are increased rates of mechanical ventilation, prolonged hospitalization durations, and escalated healthcare expenditures compared to those without IPF. Despite its limitations, our study contributes valuable insights, highlighting the need for further research on vaccination effects and emerging variants.

## Data Availability

The original contributions presented in the study are included in the article/Supplementary material, further inquiries can be directed to the corresponding author.

## Footnotes

1
https://hcup-us.ahrq.gov/db/publishing.jsp
