# Supplementary material for: Clinical impact of idiopathic pulmonary fibrosis on SARS-CoV-2 patient outcomes: a comprehensive analysis in the pre-vaccination era
Source: Front Med (Lausanne). 2025 May 21;12:1567232. doi: 10.3389/fmed.2025.1567232 (PMC12133541; doi:10.3389/fmed.2025.1567232)
Supplement: Supplementary file 1 [file Table_1.docx]

Supplementary Appendix:

**Table S1: ICD-10 codes used to cohort and stratify baseline co-morbidities.**

| **Diagnosis** | **ICD-10 CM Codes** |
| --- | --- |
| COVID-19 | U071, U00, U49, U50, U85, J1282 |
| Idiopathic Pulmonary Fibrosis | J84.112 |
| CAD | I2510, I25111, I25118, I25119, I252, I253, I254, I2541, I2542, I255, I256, I257, I2570, I25700, I25701, I2571, I25708, I25709, I25710, I25711, I25718, I25719, I25730, I25720, I25721, I25728, I25729, I2573, I25731, I25738, I25739, I2575, I25750, I25751, I25758, I25759, I2576, I25760, I25761, I25768, I25769, I2579, I25790, I25791, I25798, I25799, I258, I2581, I25810, I25811, I25812, I2582, I2583, I2584, I2589, I259 |
| DM (type 1, 2 or other) | E1100, E1101, E1137X1, E1137X2, E138X3, E1137X9, E1121, E1122, E1129, E11311, E11319, E113211, E113212, E113213, E113219, E113291, E113292, E113293, E113299, E113311, E113312, E113313, E113319, E113391, E113392, E113393, E113399, E113411, E113412, E113413, E113419, E113491, E113492, E113493, E113499, E113511, E113512, E113513, E113519, E113521, E113522, E113523, E113529, E113531, E113532, E113533, E113539, E113541, E113542, E113543, E113593, E113549, E113551, E113552, E113553, E113591, E113592, I25720, I25721, I25728, E113592, E113599, E113559, E1136, E1137, E1140, E1141, E1142, E1143, E1144, E1149, E1151, E1152, E1159, E11610, E11618, E11620, E11621, E11622, E11628, E11630, E11638, E11641, E11649, E1165, E1169, E118, E119  E0837X1, E0837X2, E0837X3, E0837X9, E0937X1, E0937X2, E0937X3, E0937X9, E0900, E0901, E0910, E0911, E0921, E0922, E0929, E09311, E09319, E093211, E093212, E093213, E093219, E093291, E093292, E093293, E093299, E093311, E093312, E093313, E093319, E093391, E093392, E093393, E093399, E093411, E093412, E093413, E093419, E093491, E093492, E093493, E093499, E093511, E093519, E093521, E093522, E093523, E093529, E093531, E093512, E093513, E093532, E093533, E093539, E093541, E093542, E093551, E093552, E093593, E093599, E0936, E093543, E093549, E093553, E093559, E093591, E093592, E0937, E0939, E0940, E0941, E0942, E0943, E0944, E0949, E0951, E0952, E0959, E09610, E09618, E09620, E09621, E09622, E09628, E09630, E09638, E09641, I25798, I25799, I258, I2581, I25810, I25811, E09649, E0965, E0969, E098, E099  E1010, E1011, E1021, E1022, E1029, E1037X1, E1037X2, E1037X3, E1037X9, E1040, E1041, E1042, E1043, E1044, E1049, E1051, E1052, E1059, E10610, E10618, E10620, E10621, E10622, E10628, E10630, E10638, E10641, E10649, E1065, E1069, E108, E109  E1300, E1301, E1310, E1311, E1321, E1322, E1329, E1337X2, E1337X1, E1337X3, E1337X9, E1340, E1341, E1342, E1343, E1344, E1349, E1351, E1352, E1359, E138, E139 |
| HTN | I10, I150, I151, I152, I158, I159, I160, I161, I169 |
| Morbid Obesity | E6601, E662, Z6835, Z6836, Z6837, Z6838, Z6839, Z6841, Z6842, Z6843, Z6844, Z6845 |
| CKD (Stage 1-5) | N181, N182, N183, N184, N185, N189 |
| ESRD | N186 |
| COPD | J44, J44.1, J44.89 J44.9 |
| Outcome | |
| Mechanical ventilation | 5A1945Z, 5A1955Z, 5A1935Z, 5A09357, 5A09457, 5A09557, 5A0935A |
| Vasopressor use | 3E030XZ,3E033XZ,3E040XZ,3E043XZ,3E050XZ,3E053XZ,3E060XZ,3E063XZ |
